# Automatic Recognition of Aggressive Behavior in Pigs Using a Kinect Depth Sensor

**DOI:** 10.3390/s16050631

**Published:** 2016-05-02

**Authors:** Jonguk Lee, Long Jin, Daihee Park, Yongwha Chung

**Affiliations:** 1 Department of Computer and Information Science, Korea University, Sejong Campus, Sejong City 30019, Korea; eastwest9@korea.ac.kr; 2 Ctrip Co., 99 Fu Quan Road, IT Security Center, Shanghai 200335, China; jinlong823@korea.ac.kr

**Keywords:** pig aggression recognition, Kinect depth sensor, support vector machine

## Abstract

Aggression among pigs adversely affects economic returns and animal welfare in intensive pigsties. In this study, we developed a non-invasive, inexpensive, automatic monitoring prototype system that uses a Kinect depth sensor to recognize aggressive behavior in a commercial pigpen. The method begins by extracting activity features from the Kinect depth information obtained in a pigsty. The detection and classification module, which employs two binary-classifier support vector machines in a hierarchical manner, detects aggressive activity, and classifies it into aggressive sub-types such as head-to-head (or body) knocking and chasing. Our experimental results showed that this method is effective for detecting aggressive pig behaviors in terms of both cost-effectiveness (using a low-cost Kinect depth sensor) and accuracy (detection and classification accuracies over 95.7% and 90.2%, respectively), either as a standalone solution or to complement existing methods.

## 1. Introduction

Recently, there has been increased interest in detecting abnormal behavior among domestic animals. If abnormal activity is not detected accurately and in a timely manner, efficient reproductive performance may be limited. Therefore, some recent studies have applied information technology to a livestock management system to minimize the damage resulting from such anomalies [1,2,3,4,5,6].

In this study, we aimed to detect and classify aggressive behaviors among weaning pigs in an intensive commercial pigsty. When unfamiliar pigs first meet after mixing group-housed weaning pigs, social conflict involving excessive aggression can occur. Aggression may be part of the behavioral repertoire that pigs exhibit to solve social conflicts [7]. When introduced to unfamiliar conspecifics, pigs naturally interact aggressively for social hierarchy status access to resources, such as space and feed [8,9,10,11]. These interactions may hamper animal welfare and increase wounding, leading to infections which may be lethal in extreme cases [12]. In addition, such aggression results in economic losses as weaker animals dominated by more aggressive ones may not have sufficient access to food so that their grow rates decrease and weight variability within the pen increases [13]. Therefore, aggression among pigs is one of the most important health, welfare, and economic problems in intensive farming [14,15].

Recently, two interesting analyses of pig aggression have been reported. First, Viazzi *et al.* [16] developed a method of detecting pigs’ aggressive behavior continuously and automatically through image processing, which enables obtaining information on the motions of pigs from historical images to find out aggressive interactions. Two features, the mean intensity of motion and the space occupation index, are derived from the segmented region of the motion history images and are used to classify aggressive interactions during episodes through linear discriminant analysis. This method was the first attempt to use image analysis to automatically detect aggressive behaviors among pigs. Second, Oczak *et al.* [17] tested a method for automatically detecting aggressive behavior in pigs using an activity index and a multilayer feed-forward neural network. In that method, the activities of the animals are measured using videos, and software is used to calculate an activity index. Five features (average, maximum, minimum, summation, and variance of the activity index) are calculated based on the video recordings, and a multilayer feed-forward neural network is trained and validated to classify events involving high and medium aggression. Their results suggest that combining the activity index with a multilayer feed-forward neural network can be used to classify aggressive pig behavior. Recently, some advances have been made in pig monitoring using red, green, and blue (RGB)-based video data; however, to the best of our knowledge, no automated analysis of anomalies using a Kinect depth sensor has been reported yet.

In contrast to current methods, in this study, we developed a non-invasive, inexpensive, and automatic monitoring prototype system that uses a Kinect depth sensor to monitor animal activity in a commercial pig facility. This proposed system notifies the farmer of an aggressive situation when it occurs in a hog barn. The method begins by extracting activity features from the Kinect depth information obtained in a pigsty. The detection and classification module, which employs two binary-classifier support vector machines (SVMs) in a hierarchical manner, detects aggressive behavior, and classifies it into aggressive sub-types such as head-to-head (or body) knocking and chasing. The results of our experiments indicate that the accuracy of aggression detection approached 95.7%, and the aggression classification approach (90.2% accuracy) was validated, where the recall and precision measures were satisfactory. As far as we know, this is the first report of aggression detection in weaning pigs by a pig monitoring system using Kinect depth data and SVMs. The results of this study suggest that Kinect depth sensors can be used to monitor the behavior of pigs. Furthermore, given the continuous and large stream of data coming from a pig monitoring system, the application of our data mining method is appropriate.

The remainder of this paper is composed as follows. Section 2 describes the proposed aggressive behavior recognition system for pigs using a Kinect depth sensor, and it provides some information on the background concepts. Section 3 presents the simulation results and Section 4 presents the conclusions.

## 2. Aggressive Behavior Recognition of Pigs Using a Kinect Depth Sensor

### 2.1. The Proposed Pig Aggression Recognition System

**Figure 1:**
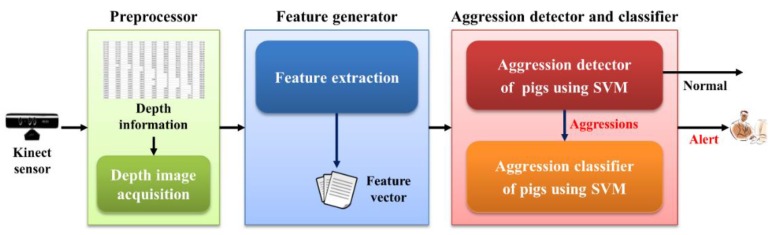
Overall structure of the pig aggression recognition system.

**Figure 2:**
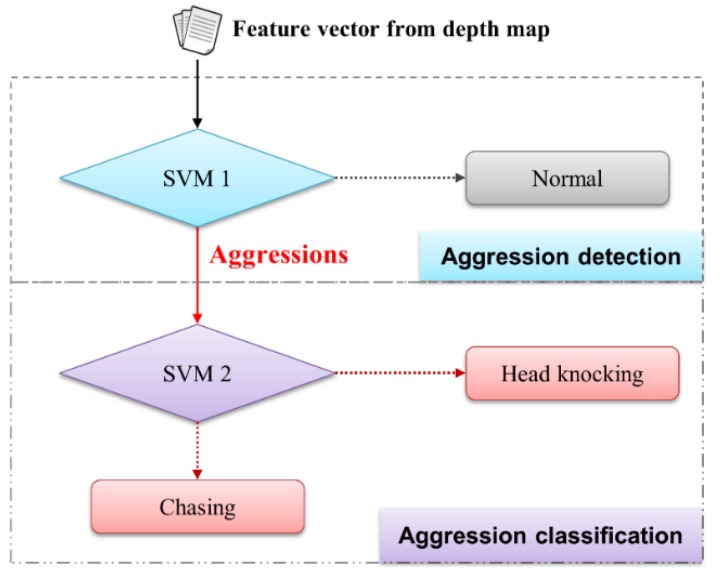
Architecture for aggression detection and classification module based on hierarchical SVM.

The proposed system for automatic detection and recognition of pig aggression consists of three modules (see Figure 1): the preprocessor, the feature generator, and the aggression detector and classifier. During preprocessing, the depth information related to pigs is obtained using a Kinect depth sensor. During the feature generation, five features (minimum, maximum, average, standard deviation of velocity, and distance between the pigs) are first extracted from the depth image. The third module uses the aggression detector and classifier to detect the aggressive behaviors, and then it classifies them hierarchically based on behavioral sub-types, such as head-to-head (or body) knocking and chasing. This study used two binary-classifier SVMs in a hierarchical manner [3,18,19,20,21]. Figure 2 shows the overall architecture of the SVM-based aggression detection and classification system.

### 2.2. Binary Classifier Support Vector Machine (SVM)

**Figure 3:**
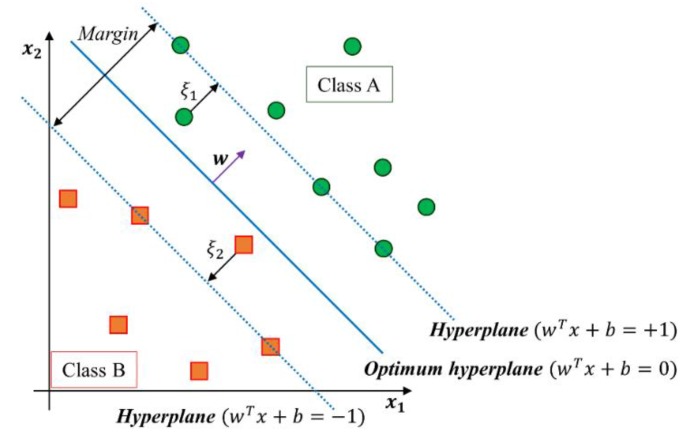
Geometric concept of the linear SVM algorithm.

The SVM is a supervised learning algorithm which was extensively used for the classification problem. The advantage of SVM is the calculation of the margin maximization that helps classification performance and strong generalization capability with limited data samples [3,18,19,20,21]. The concept of linear SVM finds the optimal hyperplane ($w^{t}x+b=0)$ to separate two classes with a maximum margin for linearly separable problem (see Figure 3).

In the linearly separable case, let $\left\{x_{1},\;x_{2},\ldots ,\;x_{z}\right\}$ be the training data set and let $y_{i}\in \left\{-1,\;+1\right\}$ be the class label of a *N*-dimensional feature vector $x_{i}$. The maximization of the margin corresponds to [3,18,19,20,21]:

(1) $$\text{min}\left[\frac{1}{2}w^{T}w+C\overset{z}{\underset{i=1}{\sum }}\xi_{i}\right]\;\;s.t.\;y_{i}\left(w^{T}x_{i}+b\right)\geq 1-\xi_{i};\;\xi_{i}\geq 0;C>0;i=1,\;\ldots ,\;z$$

Here, parameter C is a trade-off value between the training error term and the margin. Additionally, slack variable $\xi_{i}$ is a penalty for misclassification from the margin boundary or classification within the margin. When the two classes cannot be separated linearly, the approach described here for a linear SVM is needed to extend a nonlinear SVM.

**Figure 4:**
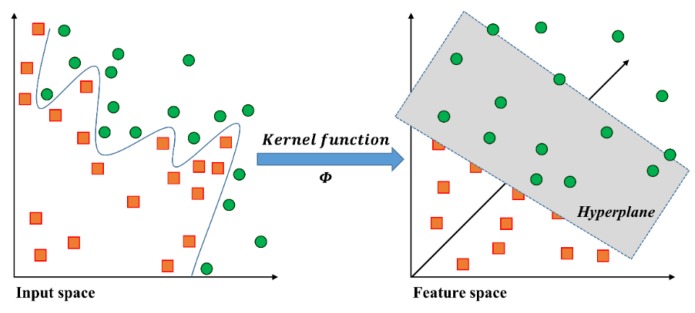
Geometric view of the nonlinear SVM.

The idea of nonlinear SVM is mapping the input training data into the higher-dimensional feature space with to the goal of obtaining linear separation (see Figure 4) [3,18,19,20,21]. In the general mathematical formulation, K is defined as the dot product of the nonlinear kernel function $K\left(x_{i},\;x_{j}\right)\equiv \phi {\left(x_{i}\right)}^{T}\cdot \phi \left(x_{j}\right)$. In particular, a radial basis function (RBF) is commonly used for the nonlinear kernel function as follows:

(2) $$K\left(x_{i},x_{j}\right)=exp(-\gamma {\left||x_{i}-x_{j}|\right|}^{2}),\;\gamma >0$$

Here, γ is a standard deviation parameter [21]. In our experiments, we employed a polynomial kernel function and an RBF kernel for detection and classification, respectively.

## 3. Results

### 3.1. Data Collection and Data Sets

**Figure 5:**
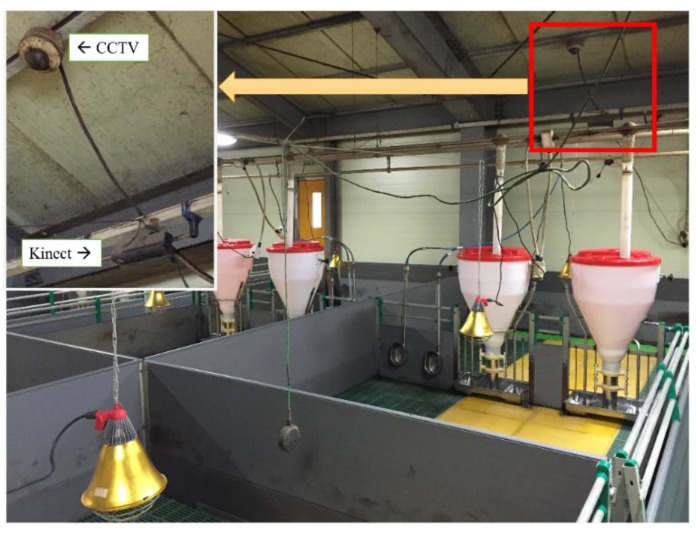
Pig housing unit installed with a stationary Kinect sensor.

**Figure 6:**
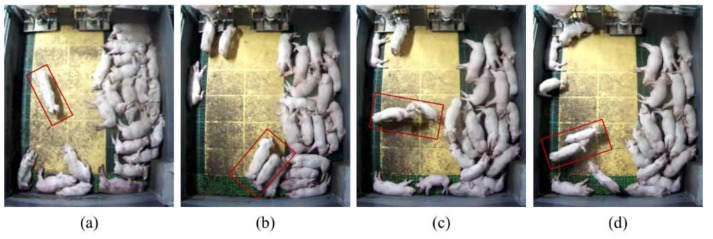
Sample images showing behaviors among the pigs: (**a**) Normal: walking alone; (**b**) Normal: walking together; (**c**) Aggression: head-to-head knocking; and (**d**) Aggression: chasing.

The experiment was conducted at a commercial swine production farm located in Sejong City, Republic of Korea. In order to collect depth videos, the pen was monitored using a top-view Kinect camera (Version 2, Microsoft, USA). Figure 5 shows a pig housing unit, complete with a stationary Kinect sensor. The pigsty contained 22 pigs ranging in weight from 25 kg to 30 kg. The resolution of a captured image was 512 × 424 pixels with a frame rate of 30 fps. Based on 50 h of recorded Kinect depth videos, 330 episodes of interactions (115 aggressive interactions: 61 head-to-head (or body) knocking, 54 chasing, and 215 normal interactions) were identified and labeled manually by a human operator. In this study, an aggressive interaction was defined as a close physical contact that lasted 1 s in which at least one of the interacting pigs exhibited head-to-head (or body) knocking or chasing behaviors. Figure 6 shows sample images of various behaviors, and the descriptions of these behaviors are summarized in Table 1.

**Figure 7:**
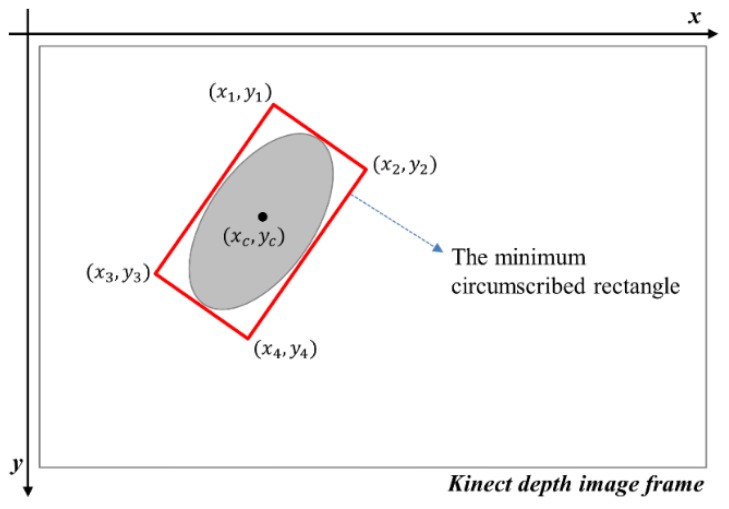
Minimum circumscribed rectangle.

**Figure 8:**
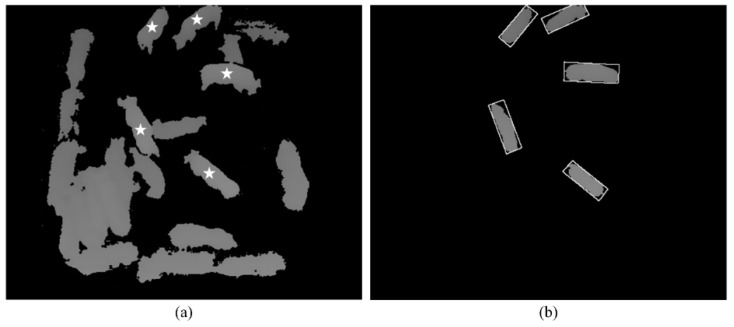
Example of pig detection based on the Kinect depth criterion: (**a**) All pigs detected by a Kinect sensor (the star symbols indicate standing pigs); (**b**) Only standing pigs detected by a Kinect sensor.

During the activity feature extraction process, the moving pigs were labeled as regions of interest (ROIs) and the barycentric coordinates of the ROIs were obtained. In general, barycentric coordinates follow piece-wise straight lines, and two coordinates for the same pig in consecutive frames are very close to each other. Thus, it is possible to measure the similarity of the two coordinates by calculating the Euclidean distance between them and determining whether they are the same based on a given threshold. Consequently, the moving pigs can be identified and tracked successfully in consecutive frames [22]. We used an index algorithm to track the pigs in the pigsty [22]. In this study, we extracted five features (minimum, maximum, average, standard deviation of velocity, and distance between the pigs) using Visual Studio 2012 (Microsoft, Redmond, WA, USA) based on the depth videos. Figure 7 shows a schematic drawing of the minimum circumscribed rectangle. In the pig activity monitoring system using a Kinect depth sensor, the pigs could be identified as either standing or lying based on the Kinect depth criterion (see Figure 8).

### 3.2. The Pig Aggression Detection and Classification Results

The aggression detection and classification module shown in Figure 1 represents the aggression detector and classifier used to detect aggressive behaviors, and it classifies them hierarchically based on aggression sub-types, such as the aggressive behaviors associated with head-to-head (or body) knocking and chasing. A personal computer (PC) (Intel^®^ i7-3770K CPU, 8 GB memory) was used to implement the proposed system, and a Weka 3.6 [23] was used. In addition, a 10-fold cross-validation was performed in all of the experiments.

First, we distinguished between aggressive and normal behaviors through an identification test conducted using the proposed method. The performance of the proposed system was evaluated via the aggression detection rate (ADR), the false positive rate (FPR), and the false negative rate (FNR) [24,25]. True positive (TP) is the aggressive behavior correctly identified as aggression; false positive (FP) is the normal behavior incorrectly identified as aggression; true negative (TN) is the normal behavior correctly identified as normal; and false negative (FN) is the aggressive behavior incorrectly identified as normal. These were determined as follows:

(3) $$ADR=\frac{TP}{TP+FN}\times 100$$

(4) $$\;FPR=\frac{FP}{FP+TN}\times 100$$

(5) $$\;FNR=\;\frac{FN}{TP+FN}\times 100$$

The detection results obtained for aggressive behavior are summarized in Table 2. The results of our experiment indicated that the detection accuracy of the proposed system was 95.7%, where FPR and FNR were 4.2% and 4.3%, respectively. As shown in Figure 2, the SVM1 detector was used in this experiment. We employed a polynomial kernel function, and the trade-off constant *C* was set to 4.5. Table 3 provides a summary of the quantitative/qualitative analysis with existing RGB-based methodologies. Note that Jin [26] proposed a method of detecting aggressive behavior in pigs using two features (mean circumscribed rectangle and velocity) extracted from the RGB-based video recordings and a SVM. In previous studies, no attempts were made to detect and classify aggressive behaviors using a Kinect depth map; thus, a performance comparison could not be conducted.

Furthermore, we classified aggressive behaviors into head-to-head (or body) knocking and chasing. The classification accuracy of the proposed system was measured using precision and recall as the performance measurements [24,25]:

(6) $$Precision=\frac{TP}{TP+FP}\times 100$$

(7) $$Recall=\frac{TP}{TP+FN}\times 100$$

The classification results obtained for the aggression behaviors analyzed in this study are summarized in Table 4. The results of our experiment indicated that the average classification accuracy was 90.2%, where the average precision and recall were 90.2% and 90.1%, respectively. The SVM 2 classifier was used in this experiment, as shown in Figure 2. We employed an RBF kernel, and the trade-off constant *C* and the Gamma value were set at 4.5 and 3.5, respectively. We also summarized the quantitative/qualitative analysis with the existing RGB-based method (in Table 5).

### 3.3. Discussion

**Figure 9:**
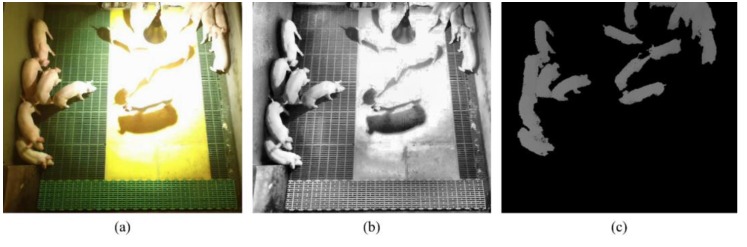
Pig detection failure due to a heating lamp in winter: (**a**) RGB input image; (**b**) CLAHE output image; and (**c**) Kinect depth image.

According to the Welfare Quality^®^ Assessment protocols, farmers should assess injuries in the pen indicating the occurrences of aggression as a way of checking the health and welfare status of their animals [27]. Since this procedure requires large amounts of time and labor, both farmers and animals should benefit from an automatic aggression monitoring system [14]. Although a rich variety of methods in behavior recognition for humans and animals using RGB video data have already been introduced in the literature [14,17,28,29,30,31,32], to the best of our knowledge, an automated analysis of anomalies for the detection of aggression in a pigsty using a Kinect depth sensor has not yet been reported. When a Kinect sensor is used, a real-time depth map can be easily captured through a simple operation and a friendly application programming interface [33]. Depth images have several advantages over RGB images: they are robust to changes in color and variations in textures, and they are robust to circumventing the problem of shadows and illumination. Depth images provide improved robustness with respect to occlusion [28,29,30,34]. In particular, controlling the hog barn temperature is one of the most important issues in pig management. However, in winter, some of the pigs captured from a RGB camera cannot be detected correctly due to the strong impact of a heating lamp. As shown in Figure 9, some areas in a pigsty cannot be detected correctly even with complicated histogram equalization techniques, such as contrast limited adaptive histogram equalization (CLAHE) [35]. In order to provide a robust solution for the problem of illumination and shadow in winter, it is more appropriate to consider the depth information. Compared to the typical stereo-camera-based solution, an infrared (IR)-based Kinect sensor can provide more accurate information at a much lower cost, without complicated camera calibration and stereo-matching operations [36].

Generally, each pig either rests or sleeps throughout most of the day and night. A single standing pig (*i.e.,* detected with a depth threshold) is also excluded from this present study’s consideration. On average, less than one interacting pig (*i.e.,* standing pigs detected as a group whose size is larger than one pig) is detected from an input depth frame, and thus needs to be classified. As explained in Section 3.1, 330 episodes of interactions were obtained from 50 h of depth videos. Therefore, real-time execution can be achieved, as shown in Table 6.

This study aimed to detect and classify aggressive behaviors among weaning pigs in an intensive commercial pigsty. Toward that end, it extracted activity features from the Kinect depth sensor information obtained in a pigsty and used two binary-classifier SVMs in a hierarchical manner to detect and classify aggressive events that were transformed into the five-dimensional feature vector space. Meanwhile, the support vector data description (SVDD) could be used to detect aggressive behaviors, as it is a natural anomaly or novelty detector in intelligent systems. Likewise, other classifiers that consider spatio-temporal features, such as the conditional random field, the recurrent neural networks, and the Markov model, are additional candidate methods that could be considered. This has yet to be investigated. As far as we know, this study is the first to report on the aggression detection of weaning pigs in a pig monitoring system using Kinect depth data and SVMs. This study’s results suggest that analysis of Kinect depth information could be a creditable method for understanding pig behavior. Because the depth map acquired from even a low-cost Kinect depth sensor can detect pig aggressive behaviors accurately and economically without causing any additional stress to the pigs, our proposed method can be used either as a standalone solution or as a way to complement other known methods in order to obtain a more accurate solution. Furthermore, even when a new aggression class appears, it can be easily adapted to our proposed system for incremental updating and scaling without reconstructing the entire system. For future work, we will consider the multi-modality of the RGB video and depth information. Our proposed system can be tested and refined further in commercial production settings, including more aggressive animal behaviors, as necessary. Thus, a complete real-time system that can incorporate the automatic detection of pig aggressive behaviors is a part of our ongoing research.

## 4. Conclusions

In the management of group-housed livestock, detecting anomalies early is very important. In particular, failure to detect aggression in a timely and accurate manner in intensive commercial pigsties could seriously limit efficient reproductive performance. In this study, we developed a low-cost, non-invasive, and automatic prototype system to monitor animal activity in a commercial pig farm, which notifies the farmer of aggression situations in the pigsty. The proposed system preprocesses an activity-feature subset by analyzing the pig activities acquired using a Kinect depth sensor. The recognition module detects aggressive behaviors and classifies them hierarchically based on aggression sub-types, such as head-to-head (or body) knocking and chasing. In our experiments, we found that the accuracy of the aggressive behavior detection obtained using the proposed system was 95.7%, and the aggressive behavior classification measures were satisfactory.

## Figures and Tables

**Table 1:** Labeled pig behaviors [17].

Aggressive Type	Behavior Label	Description
Head knocking	Head-to-head knocking	Hitting the snout against the head of the receiving pig
	Head-to-body knocking	Hitting the snout against the body of the receiving pig
Chasing	Chasing	Following another pig rapidly, usually with biting or attempted biting

**Table 2:** Aggressive behavior detection performance of the proposed system.

Aggression Detector	ADR	FPR	FNR
**SVM 1**	95.7%	4.2%	4.3%

ADR(aggression detection rate); FPR(false positive rate); FNR(false negative rate).

**Table 3:** Summary of the quantitative/qualitative analysis for the aggressive behavior detection.

Parameter	Viazzi *et al.* [16]	Jin [26]	Proposed Method
Normal data size	150	60	215
Aggressive data size	150	60	115
Used data	Private	Private	Private
Camera type	Color	Color	Depth
Camera resolution	1032×778	1280×960	512×424
Tracking	N/A	Yes	Yes
Features	Mean activity and occupation index	Mean circumscribed rectangle and velocity	Minimum, maximum, average, standard deviation of velocity, and distance between the pigs
Feature vector dimension	2	2	5
Method	Linear discriminant analysis	SVM	SVM
ADR	88.7%	93.3%	95.7%
FPR	10.7%	8.3%	4.2%
FNR	11.3%	6.7%	4.3%

**Table 4:** Performance metrics for aggressive behavior classification.

Aggression Classifier	Class	Precision	Recall
**SVM 2**	Head-to-head (or body) knocking	88.9%	92.3%
	Chasing	91.5%	87.8%
	**Average**	90.2%	90.1%

**Table 5:** Summary of the quantitative/qualitative analysis for the aggressive behavior classification.

Parameter	Oczak *et al.* [17]	Proposed Method
Aggressive behavior type	Medium and high aggression	Head-to-head (or body) knockingand chasing
Aggressive behavior data size	634/1253 seconds	61/54 episodes
Used data	Private	Private
Camera type	Color	Depth
Camera resolution	1032×778	512×424
Tracking	N/A	Yes
Features	Average, maximum, minimum, summation and variance of activity index	Minimum, maximum, average, standard deviation of velocity, and distance between the pigs
Feature vector dimension	5	5
Method	Artificial neural network	SVM
Precision	87.7%	90.2%
Recall	81.9%	90.1%

**Table 6:** Summary of the computational cost of each step.

Parameter	Execution Time (ms)	Frame Rate (fps)
Depth information acquisition (per frame)	56	17.85
Feature extraction (per interacting pigs)	10	100
Aggressive detection/classification (per episode)	1	30,000
Total *	1981 (per episode)	15.14

* Computed as one interacting-pig, assumed to be detected from one 30-framed episode.
